# A High Efficiency and Precision Smoothing Polishing Method for NiP Coating of Metal Mirror

**DOI:** 10.3390/mi13081171

**Published:** 2022-07-25

**Authors:** Chao Xu, Xiaoqiang Peng, Junfeng Liu, Hao Hu, Tao Lai, Qilin Yang, Yupeng Xiong

**Affiliations:** Laboratory of Science and Technology on Integrated Logistics Support, College of Intelligence Science and Technology, National University of Defense Technology, Changsha 410073, China; wjsxcr@126.com (C.X.); pxq2000@vip.sina.com (X.P.); laitao10@nudt.edu.cn (T.L.); qilinbit@163.com (Q.Y.); bear_xiong@163.com (Y.X.)

**Keywords:** NiP coating, SPDT, smoothing polishing, periodic marks

## Abstract

The NiP coating has excellent wear and corrosion resistance, and electroless nickel-phosphorus coating is one of the best measures for surface modification of metal optical devices. The NiP layer could be processed by single-point diamond turning (SPDT). However, the periodic marks on the surface of the NiP coating processed by SPDT will lead to diffraction and stray light, which will reduce the reflectivity and image quality of the mirror. This paper studied smoothing polishing based on chemical mechanical polishing to remove turning periodic marks efficiently. Firstly, we studied the chemical corrosion and mechanical removal mechanism of smoothing polishing of the NiP coating through theoretical analysis. Then, the influencing factors of processing the quality of smoothing polishing are analyzed, and the optimal machining parameters and polishing slurry formula are formulated. Finally, through the developed process, the surface roughness of Root Mean Square (RMS) 0.223 nm is realized on the NiP coating, and an ultra-smooth surface that can meet the service accuracy of a hard X-ray mirror is obtained. Our research simplifies the high-precision machining process of the NiP coating and improves the machining efficiency. Therefore, it can be used as a new high-precision manufacturing NiP coating method.

## 1. Introduction

Metallic materials exhibit unique advantages in optics application due to their good characteristics [1,2]. We could use metal to process mirrors and structural supports of optical systems [3,4]. Structural supports and mirrors can be made from the same material to avoid thermal stress due to different CTEs. The optical systems can achieve the athermal design when the mirrors and system supporting structure elements are made of the same metal material. It is significant for applications with high lightweight requirements and temperature-variable environments, such as aerospace [5]. We can optimize the entire process of optical system design, manufacturing, testing, assembly, adjustment, and realize optical-mechanical integrated manufacturing by using metal optical components. Therefore, the optical systems with metal elements have advantages with a more compact structure, shorter development and production cycle, and higher product cost performance [6,7]. According to the calculation of Raytheon, the manufacturing cost of metal mirrors is much lower than ceramics and glass mirrors [8]. The European Space Agency (ESA) has also comprehensively promoted the application of metal mirrors in visual remote sensing loads below 500 mm [9]. Therefore, the metal optical system has broad market prospects.

The metal materials for making mirrors are mostly aluminum and beryllium. However, beryllium is toxic, and a series of protective measures are taken during processing, which greatly increases the cost, so the application of beryllium is limited [10]. Aluminum has the advantages of a low price, good machinability, low density, high lightweight rate, and manufacturing complex curved optical components [11,12]. Nevertheless, aluminum contains impurities, which are prone to surface defects during processing and affect the surface quality [13]. The typical range of micro-roughness that can be achieved by processing aluminum mirrors is RMS 2 nm to RMS 10 nm, which can be used in infrared spectroscopy only and is difficult to meet the requirements of shorter wavelength ranges. The roughness should be better than RMS 1 nm for mirrors to be suitable in the visible spectral range [2]. Moreover, in higher demand, such as hard X-ray mirror applications, the micro-roughness must be better than RMS 0.3 nm [14].

The NiP coating has excellent machinability due to its uniform and dense material properties. Therefore, the NiP coating is commonly used for surface modification of metal mirrors to improve processing performance [15,16]. Consequently, we could obtain high-quality metal optical components by processing the NiP coating [17,18]. SPDT is the most common method for machining metal optical devices, which could directly receive surfaces that meet the quality requirements of infrared imaging [19]. However, the machining accuracy of SPDT is restricted by the accuracy of the lathe. In addition, the SPDT would produce periodic marks, which would lead to diffraction and stray light, increasing the loss of luminous flux and decreasing the reflectivity and image quality [20,21]. In consequence, after turning the NiP coating with SPDT, further machining is required to meet higher demands.

Many polishing techniques are used to eliminate periodic marks of the NiP coating, such as magnetorheological polishing [22], float polishing [14], and fluid jet polishing [23]. Magnetorheological polishing is mainly used to correct the surface shape error, and the ability to improve the surface quality is insufficient. Yang Bai polished the NiP coating on the aluminum substrate by magnetorheological polishing, and the surface quality was only RMS 1.046 nm [22]. Among the reported processing methods of the NiP coating, the surface quality achieved by float polishing is the highest. However, float polishing has the defect of low efficiency [14]. Fluid jet polishing could eliminate turning marks of the NiP coating, but its ability to improve the surface quality is insufficient also. Anthony Beaucamp used fluid jet polishing to process the NiP coating and obtained the surface roughness of RMS 1.689 nm. Subsequently, it improved the surface quality through bonnet polishing, which increases the process complexity [23]. In summary, the disadvantage of magnetorheological and fluid jet polishing is the poor quality of the polished surface, and the weakness of float polishing is inefficiency.

Chemical mechanical polishing (CMP) is a surface treatment method that uses the joint action of chemical corrosion and abrasive removal [24,25]. It is one of the key processes to achieving the performance goals of modern microprocessors and memory chips [26]. However, the chemical mechanical polishing technology used in semiconductor processing is a global planarization technology, which cannot realize the processing of large aperture and nonplanar mirrors. The smoothing polishing is based on the principle of chemical mechanical polishing, and the size of the polishing disc used in polishing is 1/4 to 1/16 of the size of the optical element [27]. Smoothing polishing also has a unique combination of chemical and mechanical action and is an effective way to remove turning marks.

This paper proposes a smoothing polishing method for the NiP coating, which can effectively remove the periodic marks of SPDT and provide a new idea for the high-precision manufacturing of metal mirrors. First, in Section 2, the key factors affecting smoothing polishing are analyzed theoretically, creating conditions for the high-precision fabrication of NiP coatings. Then, an experiment was carried out in Section 3. Finally, we analyzed the experimental results in Section 4. Through verification, the smoothing polishing method developed by our study has excellent stability.

## 2. Materials and Methods

### 2.1. Materials

**Figure 1:**
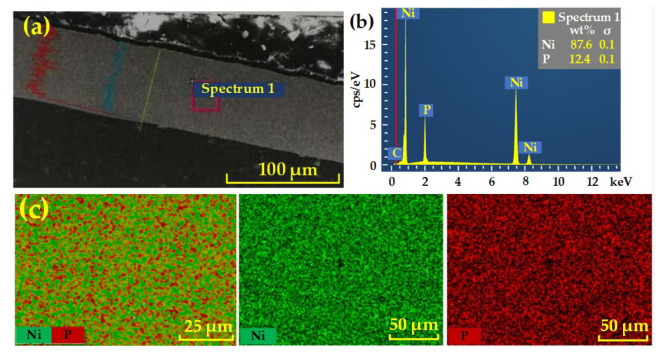
Test results of the experimental sample. (**a**) Cross-sectional morphology of the NiP coating, (**b**) Elemental analysis result, and (**c**) EDS mapping.

The NiP coating was deposited on the aluminum alloy substrate by electroless plating. The performance of the NiP coating would be affected by the structure, phosphorus content, and thickness of the coated layer. In practical applications, NiP coatings need a uniform and dense microstructure to obtain high surface quality through ultra-precision machining. An electroless NiP coating with low phosphorus content has a high melting point and crystal structure. In contrast, the high phosphorus coating forms an amorphous structure, which is a non-magnetic coating, and the hardness of the layer also decreases with the increase of phosphorus content [28,29]. At the same time, it is also necessary to pay attention to the thickness of the coating. Insufficient coating thickness would cause the layer to be damaged during processing. The sample for our experiment was examined by scanning electron microscope and energy dispersive spectrometer from Carl Zeiss, Germany. The detection results are shown in Figure 1. The layer is uniform and dense. The fraction of Ni in the coating is 87.6 wt%, the fraction of P is 12.4 wt%, and the thickness of the coating is about 80 μm.

### 2.2. Material Removal Mechanism of NiP Coating Based on Smoothing Polishing Machining

**Figure 2:**
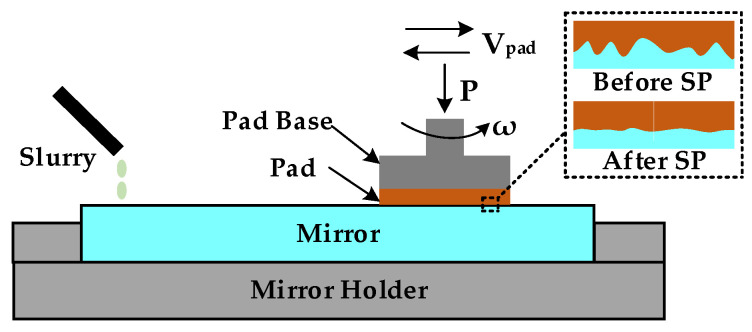
Schematic of the smoothing polishing.

Smoothing polishing (SP) is a surface finishing process based on chemical mechanical polishing, in which chemical corrosion and abrasive polishing work together. As shown in Figure 2, smoothing polishing requires equipment, a polishing pad, a polishing slurry, etc. During polishing, the polishing pad maintains good contact with the workpiece to be processed under the action of the cylinder pressure. The polishing pad rotates around its center and moves on the workpiece according to the set path.

Through smoothing polishing, we could effectively remove the periodic marks caused by SPDT without deteriorating surface forms. The essence of smoothing polishing is to form an easily removable oxide layer on the surface of the workpiece by chemical reaction and then produce mechanical removal by using the positive pressure and relative movement of the polishing abrasive. The polishing pressure and the speed of the polishing disc are the key process parameters. The greater the pressure and the rate of the polishing disc during polishing, the easier the abrasive particles are to press into the workpiece and cause surface scratches [30]. Therefore, it is necessary to study the influence of polishing slurry on chemical action and the effect of polishing parameters on mechanical removal. According to these requirements, we could optimize the formulation of the polishing slurry, the speed of the polishing pad, and the polishing pressure to improve the polishing process.

The polishing slurry in our study is composed of silica, hydrogen peroxide, complexing agent, pH regulator, and deionized water. In the process of smoothing polishing, the oxidant in the slurry reacts with Ni and P to form a thin oxide layer on the surface, which is removed by the mechanical action of abrasive particles later. If the removal of the oxide layer is not timely, it will affect the continuation of the oxidation reaction and reduce the material removal rate. On the other hand, we could obtain the highest material removal efficiency and the best surface quality when the coordination between the chemical reaction and abrasive mechanical removal is the best. Therefore, studying the chemical reaction mechanism between the oxidant and the NiP coating is necessary.

**Figure 3:**
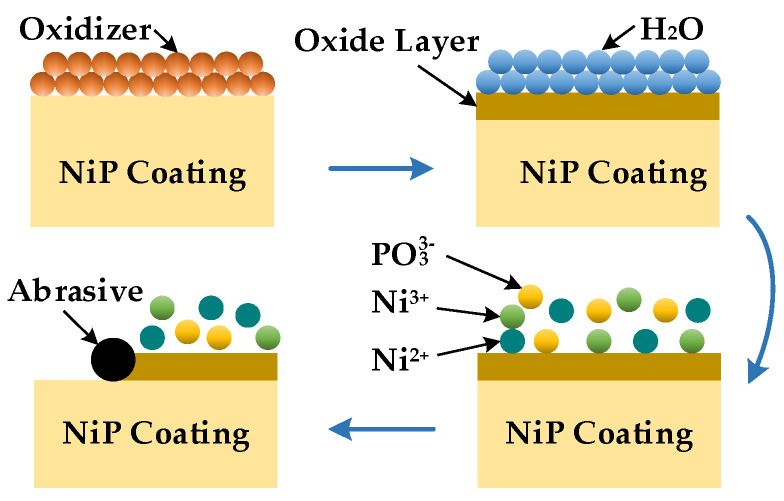
Schematic diagram of the chemical reaction process.

The chemical reaction process is shown in Figure 3 [31]. With hydrogen peroxide (H_2_O_2_) as an oxidant, the NiP coating generates a thin oxide film on the surface under the action of the oxidant. The reaction equation is Equations (1)–(3).

(1) $$Ni+H_{2}O_{2}\to NiO+H_{2}O$$

(2) $$NiO+H_{2}O_{2}\to Ni_{2}O_{3}+H_{2}O$$

(3) $$P+H_{2}O_{2}\to P_{2}O_{3}+H_{2}O$$

From the above reaction formula, the surface of the NiP coating is oxidized and forms a mixture of NiO, Ni_2_O_3_, and P_2_O_3_ under the action of hydrogen peroxide in the polishing slurry. In the polishing slurry, phosphorus oxide will be hydrolyzed, and nickel oxide will react with water to form nickel hydroxide. The reactions are as follows:

(4) $$P_{2}O_{3}+H_{2}O\to H_{3}PO_{3}$$

(5) $$NiO+H_{2}O⇄Ni{\left(OH\right)}_{2}⇄Ni^{2+}+OH^{-}$$

(6) $$Ni_{2}O_{3}+H_{2}O⇄Ni{\left(OH\right)}_{3}⇄Ni^{3+}+OH^{-}$$

On the one hand, Ni^2+^ and Ni^3+^ produced by the reaction can react with the complex after entering the slurry, making the reaction tend to the right and accelerating the oxidation reaction. On the other hand, the adhesion between the oxide film and the NiP coating is less than that between the internal molecules of the coating, so the oxide film is easier to remove. At the same time, the micromorphology of the coating surface is undulating, and the material removal rate at the bulge is faster than depression. In addition, the oxide film at the depression can play a protective role and avoid processing damage. Therefore, oxidant and complexing agents can improve the polishing efficiency and help obtain a smoothing surface.

**Figure 4:**
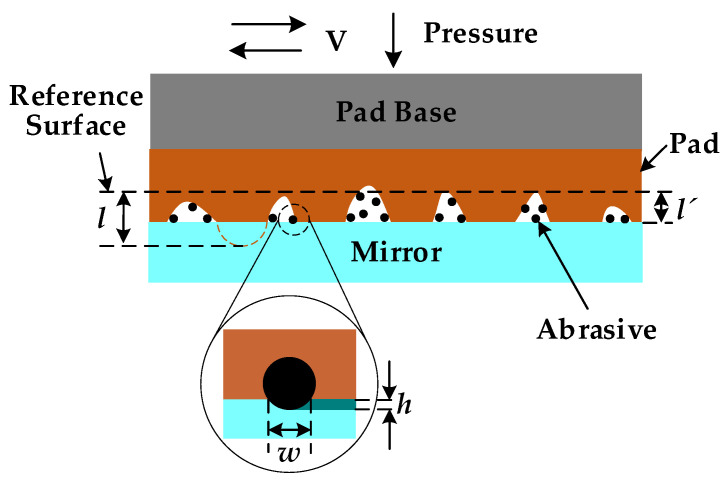
Schematic diagram of mechanical action.

The polishing pad in the smooth polishing of the NiP coating is made of asphalt. Asphalt is a kind of viscoelastic material with a soft texture, and abrasive particles are easy to be embedded in it. The polishing pad drives the abrasive particles across the surface to remove the material during polishing. Therefore, it is necessary to study the contact state among abrasive particles, workpieces, and polishing pads to establish a material removal model. As shown in Figure 4, the contact between the abrasive particles and the workpiece is small deformation contact, so the material removal modeling can be carried out according to the small deformation of contact mechanics theory and the force balance principle. The macroscopic material removal rate equals the material removal rate of a single abrasive particle multiplied by the number of abrasive particles. To simplify the modeling process, we only establish the model of single-particle material removal between polishing disc, abrasive particle, and workpiece.

According to the theory of contact mechanics [32], we can approximately consider that the workpiece is a flat surface, and the polishing disc is a nominally flat surface. The abrasive particles on the polishing disc contact the workpiece directly. We can express contact force *F_ap_* between the polishing pad and the abrasive particles as:

(7) $$F_{ap}=\frac{4}{3}E_{p}{\left(\frac{d}{2}\right)}^{\frac{1}{2}}{\left(d-h\right)}^{\frac{3}{2}}$$

where *E_P_* is the elastic modulus of the polishing pad; *d* is the diameter of the abrasive; *h* is the pressing number of abrasive particles on the workpiece. We can express contact force *F_am_* between the workpiece and abrasive as:

(8) $$F_{am}=\pi H_{m}dh$$

where *H_m_* is the following formula that can approximately express the hardness of the workpiece, section area of removed area Δ*S*:

(9) $$\Delta S\approx \frac{2}{3}hw=\frac{4}{3}h\sqrt{{\left(\frac{d}{2}\right)}^{2}-{\left(\frac{d}{2}-h\right)}^{2}}=\frac{4}{3}\sqrt{dh^{3}}$$

Based on the above analysis, the removal rate *RR_V_* of a single abrasive particle can be expressed as:

(10) $$RR_{V}=\frac{Vt\Delta S}{t}=\frac{4}{3}V\sqrt{dh^{3}}$$

Under the stable polishing state, the force balance state is reached among the abrasive particles, workpiece, and polishing pad, and *h* can be calculated by equating Equation (7) = Equation (8).

According to the above analysis, the material removal of smoothing polishing of the NiP coating is related to the type and content of polishing abrasive particles, oxidant, complexing agent, pH value of the polishing solution, polishing pressure, and polishing speed. Under the existing conditions, accurately predicting the material removal rate in the polishing process is impossible. Still, we can determine the main factors affecting the removal rate according to the analysis to provide theoretical guidance for the experiment.

In the smoothing polishing of the NiP coating, the most important thing is to balance the mechanical removal and the chemical reaction in the polishing process to obtain a high-quality surface and improve the processing efficiency. Therefore, the research in Section 3 focuses on balancing the mechanical removal and chemical reaction in the polishing process to optimize the process parameters and the formulation of the polishing slurry.

## 3. Experiment Setup

### 3.1. Single-Point Diamond Turning (SPDT)

In practice, the SPDT turning is the entry process to obtain a certain initial surface shape and quality. In our experiment, the workpiece was a flat mirror with an NiP coating on Al6061 with a diameter of 30 mm. We use an SPDT lathe (Precitech Nano form 350) to turn the NiP coating to achieve a mirror effect on the surface. The turning parameters are shown in Table 1.

### 3.2. Preparation of Polishing Slurry

Smoothing polishing is carried out on the CNC small grinding head machine tool (self-made by the research group). The equipment could polish plane workpieces and curved surface workpieces. The maximum stroke of the equipment′s linear axis X and Y is 650 mm, the repeatability of positioning is 0.01 mm/500 mm, and the full stroke of the linear axis Z is 200 mm. The repeated positioning accuracy is 0.008 mm/500 mm. The repeated positioning accuracy of the rotary axes A, B, and C ≤ ± 20″, and the maximum travel of the A and B axes is ±30°, and the full travel of the C axis is *n* × 360°.

**Figure 5:**
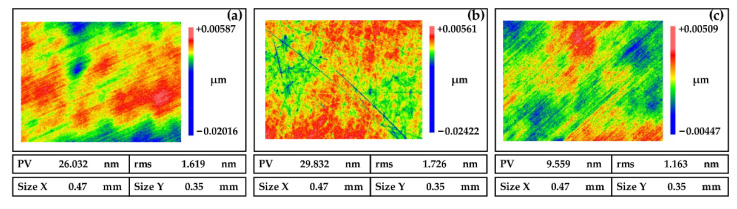
Polished surfaces of NiP coating induced by different abrasive (**a**) CeO_2_, (**b**) Al_2_O_3_, and (**c**) SiO_2_.

Abrasives play a role in material removal in polishing, so selecting the appropriate type of abrasives is very important. In chemical mechanical polishing, the wide application abrasives are CeO_2_, SiO_2_, and Al_2_O_3_. As shown in Figure 5a (measured by white light interferometer (Zygo NewView 700) under a 20× lens with a scan size of 0.47 mm × 0.35 mm), the material removal rate of the NiP coating is low due to the low hardness of CeO_2_, which affects the processing efficiency. As shown in Figure 5b, the hardness of the NiP coating is less than that of Al_2_O_3_, and scratches are easily generated during polishing, which affects the surface quality. SiO_2_ has moderate hardness and could maintain long-term stability in the polishing slurry without agglomeration. As shown in Figure 5c, polishing NiP coatings with SiO_2_ enables good surface quality. Therefore, SiO_2_ is selected as the polishing abrasive.

**Figure 6:**
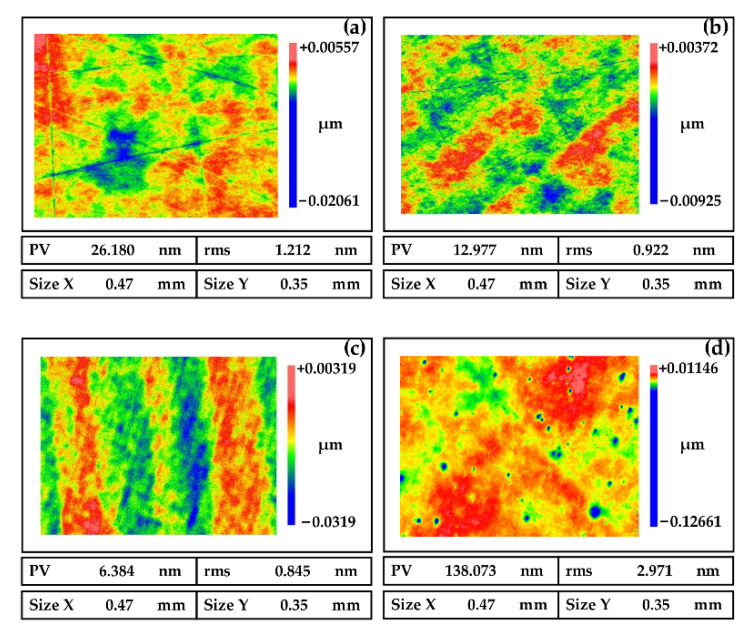
Polished surfaces of NiP coating induced by oxidants of different concentrations (**a**) 0 wt%, (**b**) 5 wt%, (**c**) 10 wt%, and (**d**) 15 wt%.

The main function of the oxidant is to react with the surface of the NiP coating to form an oxide layer that is easily removed by mechanical action. Therefore, the composition and content of the oxidant would affect the polishing effect. Hydrogen peroxide, ferric nitrate, potassium permanganate, and sodium hypochlorite are widely used oxidants. Among them, hydrogen peroxide is non-toxic and non-polluting, does not cause harm to people and the environment, and does not pollute the surface with metal ions, so hydrogen peroxide is selected as the oxidant. From Figure 6a,b, we can see that the mechanical removal of abrasive particles is greater than the chemical corrosion effect with low oxidant content, which leads to scratches on the surface. On the other hand, from Figure 6d, pits are easily formed on the surface with high oxidant content. Therefore, the content of the oxidant needs to be moderate, as shown in Figure 6c.

The use of complexing agents affects the polishing rate. The complexing agent controls the concentration of free nickel ions entering the polishing slurry, which can improve the stability of the slurry. We choose hydroxy-containing fruit acid as the complexing agent. Fruit acid is non-toxic and non-polluting and will not cause harm to people and the environment. In addition, the hydroxyl groups and nickel ions in the fruit acid can form stable nickel ligands, which can promote Equations (4)–(6) to proceed to the right and increase the reaction rate.

**Figure 7:**
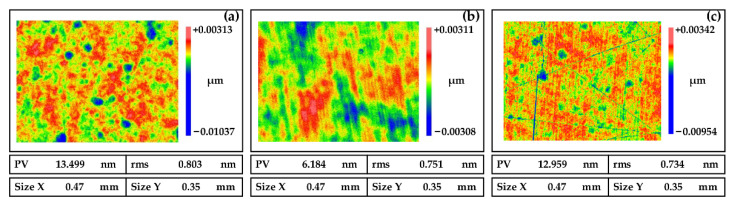
Polished surfaces of NiP coating induced by slurries with pH values at (**a**) 3.5, (**b**) 6.5, and (**c**) 9.5.

In the chemical reactions analyzed in Section 2.2, the pH value of the slurry is a key factor affecting the stability of the chemical reaction. Figure 5 depicts the polished surface morphology of NiP coatings at different pH values. There are pitting pits in Figure 7a, no obvious machining defects in Figure 7b, and a lot of scratches in Figure 7c. The chemical reaction is violent under acidic conditions, and pitting pits are generated by acid etching. Under alkaline conditions, the polishing slurry crystallizes, and the grain size is much larger than that of polishing abrasive grains. During polishing, the scratch occurs due to the grain sliding on the surface. Therefore, it should stabilize the pH value of the polishing slurry at around 6.5.

In the above analysis, we optimized the polishing slurry’s abrasive type, oxidant content, and pH value. The optimization range is shown in Table 2.

According to the above analysis and referring to related research [31], we developed a polishing slurry for smoothing polishing of the NiP coating, which consists of an oxidizing agent (H2O2), abrasive (SiO2), complexing agent, pH adjusting agent, and deionized water. This polishing slurry is non-toxic and does not damage operators’ health. In addition, the fluid’s pH value is close to neutral, which does not damage the processing equipment. The specific content of the slurry is shown in Table 3. The No. 1 polishing slurry is used to remove the periodic structure from SPDT quickly, and the No. 2 polishing slurry is used to improve the surface quality further.

### 3.3. Optimize Polishing Parameters

**Figure 8:**
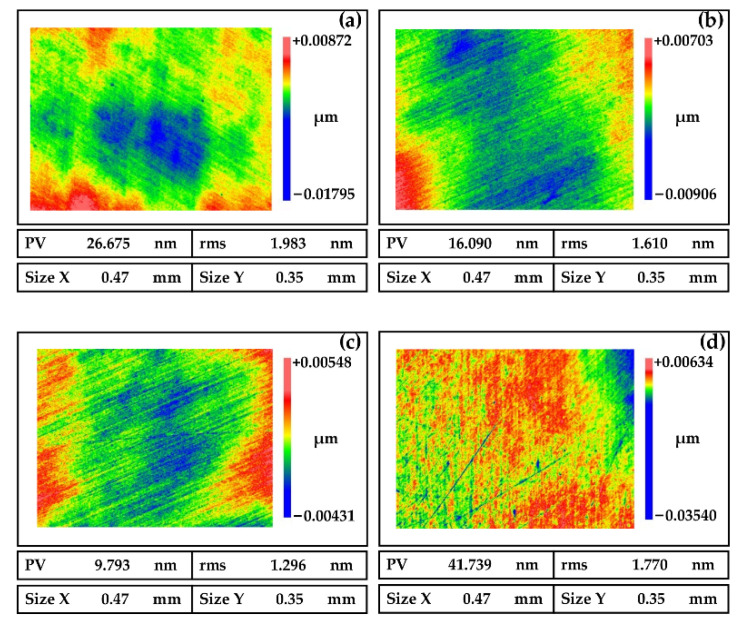
Polished surfaces of NiP coating induced by different pressures (**a**) 0 MPa, (**b**) 0.01 MPa, (**c**) 0.02 MPa, and (**d**) 0.03 MPa.

Polishing pressure is one of the important factors affecting NiP coating polishing. As shown in Figure 8a,b, the surface roughness decreases with the increase of the polishing pressure when the polishing pressure is less than 0.02 MPa. Conversely, from Figure 8c,d, we can see that the surface roughness deteriorates rapidly with the rise of the polishing pressure when the polishing pressure is more than 0.02 MPa. This phenomenon is because the friction between the workpiece and the abrasive particles is small when the polishing pressure is too small and the material removal rate is low. At this time, the mechanical action is less than the chemical action, so we cannot obtain a good surface quality. On the other hand, if the polishing pressure is too large, the abrasive particles are easily embedded in the surface of the workpiece, which is prone to surface scratches.

**Figure 9:**
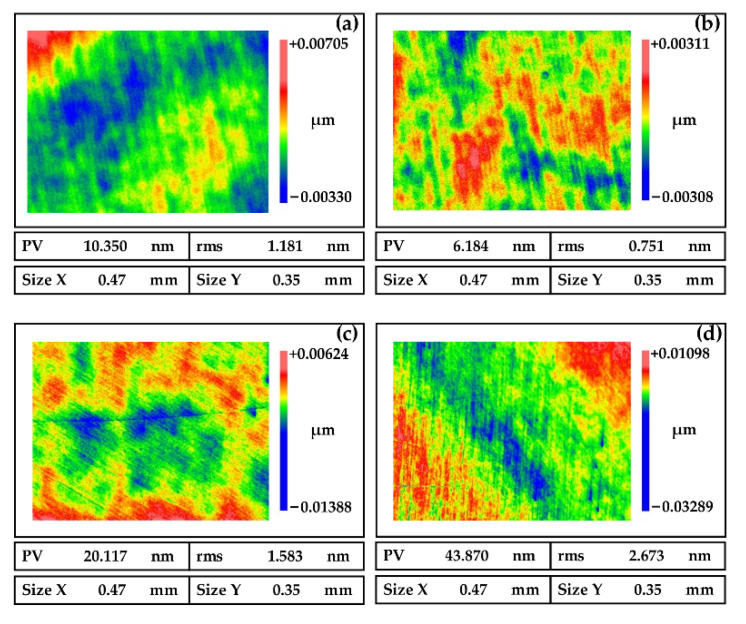
Polished surfaces of NiP coating induced by different speeds (**a**) 50 rpm, (**b**) 100 rpm, (**c**) 150 rpm, and (**d**) 200 rpm.

As shown in Figure 9a,b, the surface roughness decreases with the increase of polishing disc rotation speed when the polishing rotation speed is less than 100 rpm. Conversely, from Figure 9c,d, we can see that the surface roughness increases with polishing rotation speed when the polishing pressure is greater than 150 rpm. This phenomenon is because the fluidity of the polishing liquid is small when the rotation speed is too low. The abrasive particles are participating in the polishing decrease, reducing the material removal rate. On the other hand, the centrifugal force is too large when the rotation speed is too high, which would reduce the polishing liquid involved in the processing and reduces the material removal rate, affecting the surface quality.

The feed speed of the small grinding head polishing machine tool refers to the scanning speed of the polishing disc. Its influence on the polished surface quality is the same as that of the speed of the polishing disc on the polished surface quality. With the feed speed increase, the polished surface’s roughness decreases first and then increases. Therefore, we are not repeating it here.

In the above analysis, we optimized the polishing pressure, polishing disc rotation speed, and feed speed among the processing parameters. The optimization range is shown in Table 4.

Through the above analysis, we optimized the polishing process of the NiP coating and selected the appropriate processing parameters. The processing parameters are shown in Table 5. The No. 1 polishing parameter can quickly remove the periodic turning structure, and the No. 2 polishing parameter is used to improve the surface quality.

## 4. Results and Analysis

### 4.1. SPDT Process

**Figure 10:**
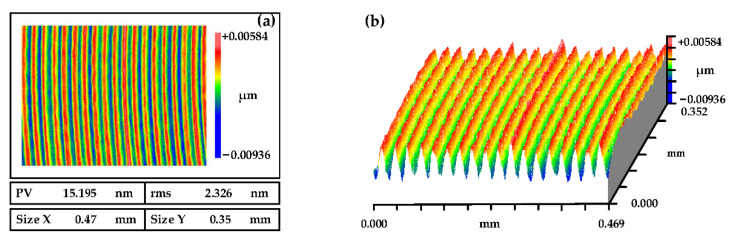
The roughness of the SPDT process. (**a**) Measurement Results, and (**b**) Three-dimensional topography.

The surface morphology of the NiP coating through SPDT is shown in Figure 10. It was measured by a white light interferometer (Zygo NewView 700) under a 20× lens, the scanning size was 0.47 mm × 0.35 mm, and the surface roughness was RMS 2.326 nm. From Figure 10, we can see that the surface presents an obvious periodic structure, which would enhance the surface scattering effect, increase the loss of light flux, cause dispersion in the optical system and deteriorate the visual performance of the component. Therefore, subsequent processing must be carried out to improve the surface quality from SPDT.

### 4.2. Smoothing Polishing Process

**Figure 11:**
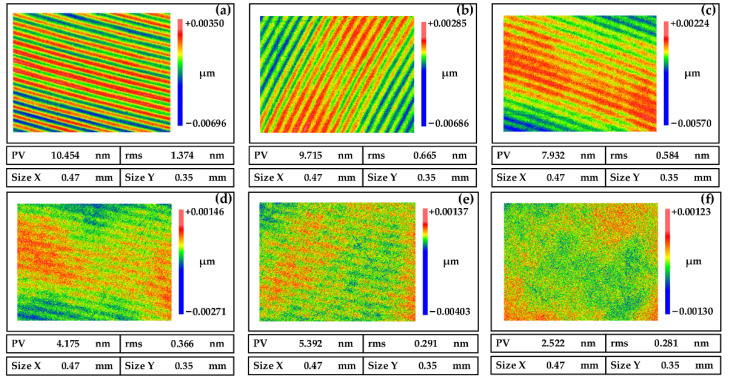
The roughness of the smoothing polishing process. (**a**) First polishing, (**b**) Second polishing, (**c**) Third polishing, (**d**) Fourth polishing, (**e**) Fifth polishing, and (**f**) Sixth polishing.

The NiP coating was polished six times, as shown in Figure 11. We use the process parameters of No. 1 in Table 3 and the formula of the polishing slurry of No. 1 in Table 5 for the first four polishing times. Each polishing takes 4 min to achieve the purpose of quickly smoothing the periodic marks. Next, we use the parameters of No. 2 in Table 3 and the formula of the polishing solution No. 2 in Table 5 for the last two polishing times. The polishing time was 6 min each time. Finally, we obtained the high-quality NiP coating surface with a surface roughness of RMS 0.281 nm.

**Figure 12:**
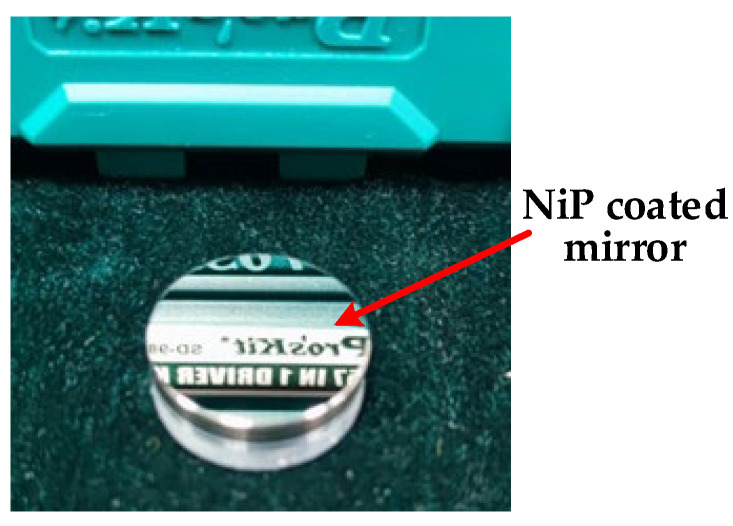
NiP-coated mirror with smoothing polishing.

The mirror through smoothing polishing is shown in Figure 12. Again, we could observe that the surface of the workpiece is bright, and there are no surface defects such as scratches.

### 4.3. Verification of Process Stability

**Figure 13:**
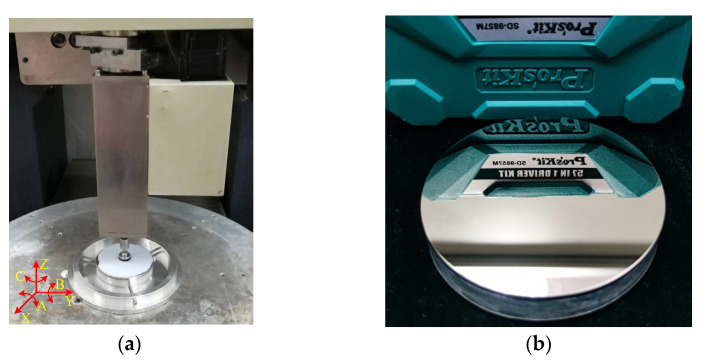
Φ100 mm mirror (**a**) smoothing polishing, and (**b**) finished mirror.

We performed processing on a mirror with a diameter of Φ100 mm to verify the process stability of smoothing polishing, as shown in Figure 13. The thickness and phosphorus content of the NiP coating are the same as those of the NiP coating flat mirror with a diameter of 30 mm in Section 3.1.

**Figure 14:**
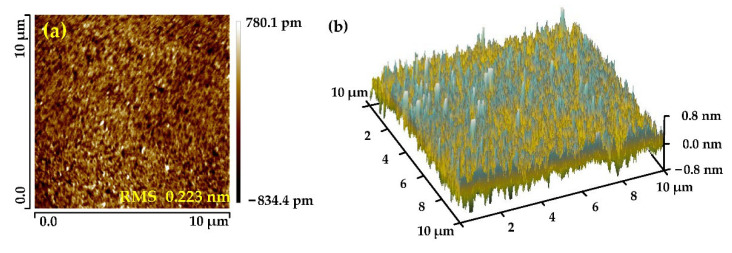
Surface roughness of Φ100 mm mirror after polishing (**a**) Measurement Results, and (**b**) Three-dimensional.

The surface roughness of the NiP coating was measured by atomic force microscope (Dimension Icon, Bruker, Rheinstetten of Germany) after six times of smoothing polishing. The results are shown in Figure 14, the surface roughness of the NiP coating reached RMS 0.223 nm.

**Figure 15:**
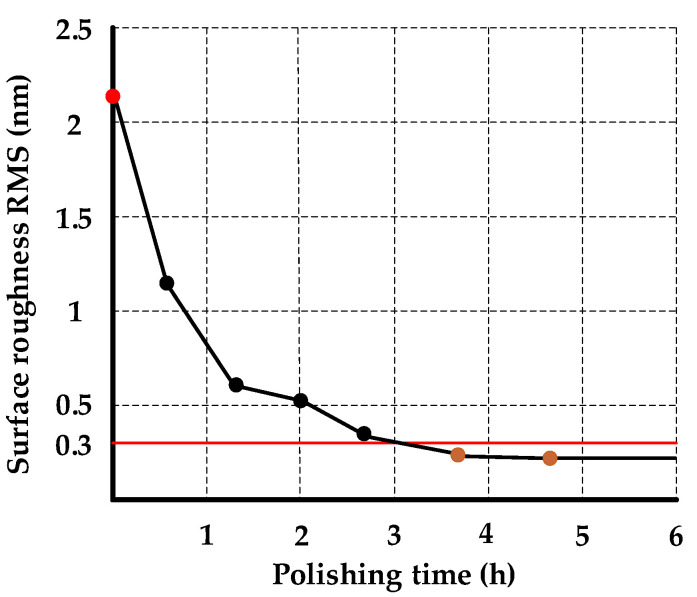
Surface roughness variation of NiP coating during polishing.

Figure 15 shows the change process of the surface roughness of the NiP coating during smoothing polishing. After 4.5 h of smoothing polishing, the surface roughness of the layer reached RMS 0.223 nm. This paper’s polishing slurry and processing technology can obtain the surface roughness below RMS 0.3 nm when processing the NiP coating.

The surface roughness of the NiP coating reaches RMS 0.223 nm through smoothing polishing, which indicates that the chemical action and mechanical removal in polishing have achieved a great balance state. Furthermore, it takes less than 5 h to process a Φ100 mm mirror, which shows high processing efficiency. At the same time, we tested the pH value of the polishing slurry after polishing. We found that the pH value of the polishing slurry increased by about 0.4 (through the comparative experiment, we excluded the influence of water evaporation in the polishing slurry). From Equations (5) and (6) in Section 2.2, we can find that the hydroxide of Ni has an ionization balance in the slurry, and the OH^−^ produced by the hydrolysis reaction will raise the pH of the slurry. Therefore, it is necessary to take measures to stabilize the pH value of the polishing slurry.

## 5. Conclusions

With the increasing application of metal optical devices, the NiP coating has a broad development prospect because of its excellent processing and optical properties. Firstly, the material removal mechanism of smoothing polishing is studied in this paper, which provides a theoretical basis for realizing the balance between mechanical and chemical effects. Then, we carried out the polishing test and selected the appropriate polishing abrasive, oxidant, and complexing agent, optimized the key machining parameters such as pressure, rotating speed, and feed rate. Finally, a high-precision smooth polishing process of the NiP coating is formed. In the experiment, we found that:

Nickel oxides would react with water to form nickel hydroxides during the polishing process, which have a weak ionization balance in the water. Therefore, the pH of the polishing slurry would increase by about 0.4 as the polishing progresses. This increase would adversely affect the polishing quality, so it is necessary to take measures to stabilize the pH of the polishing slurry.

A surface roughness below RMS 0.3 nm can be obtained on the NiP coating based on the proposed smoothing polishing method, and it only takes 28 min to machine a workpiece with a diameter of 30 mm. The goal of high-precision and -efficiency machining has been achieved.

## Figures and Tables

**Table 1:** Parameters of SPDT process.

Spindle Speed (rpm)	Feed Rate (mm/min)	Depth of Cut (μm)	Tool Nose Radius (mm)
1000	1.5	1.5	1.027

**Table 2:** Optimization range of polishing slurry composition.

Abrasive (wt%)	Oxidizer (wt%)	Complexing Agent (wt%)	pH
5~20	0~15	0~10	3.5~9.5

**Table 3:** Composition of the polishing slurry.

No.	Abrasive (wt%)	Oxidizer (wt%)	Complexing Agent (wt%)	pH
1	15	10	5	6.5
2	12	8	4	6.8

**Table 4:** Optimization range of processing parameters.

Pressure (MPa)	Rotation Speed (rpm)	Feed Rate (mm/min)
0~0.03	50~200	50~200

**Table 5:** Parameters of smoothing polishing.

No.	Pressure (MPa)	Rotation Speed (rpm)	Feed Rate (mm/min)
1	0.020	120	150
2	0.015	90	100

## Data Availability

Not applicable.
